# Shear Behavior Models of Steel Fiber Reinforced Concrete Beams Modifying Softened Truss Model Approaches

**DOI:** 10.3390/ma6104847

**Published:** 2013-10-23

**Authors:** Jin-Ha Hwang, Deuck Hang Lee, Hyunjin Ju, Kang Su Kim, Soo-Yeon Seo, Joo-Won Kang

**Affiliations:** 1 Department of Architectural Engineering, University of Seoul, 163 Seoulsiripdae-ro, Dongdaemun-gu, Seoul 130-743, Korea; E-Mails: jinhahwang@uos.ac.kr (J.-H.H.); dklee@uos.ac.kr (D.H.L); fis00z@uos.ac.kr (H.J.); 2 Department of Architectural Engineering, Korea National University of Transportation, 50 Daehak-ro Chungju-si, Chngbuk 380-702, Korea; E-Mail: syseo@ut.ac.kr; 3 School of Architecture, Yeungnam University, 280 Daehak-ro, Gyeongsan-si, Gyeongbuk 712-749, Korea; E-Mail: kangj@ynu.ac.kr

**Keywords:** steel fiber, SFRC, shear behavior, shear strength, softened truss model

## Abstract

Recognizing that steel fibers can supplement the brittle tensile characteristics of concrete, many studies have been conducted on the shear performance of steel fiber reinforced concrete (SFRC) members. However, previous studies were mostly focused on the shear strength and proposed empirical shear strength equations based on their experimental results. Thus, this study attempts to estimate the strains and stresses in steel fibers by considering the detailed characteristics of steel fibers in SFRC members, from which more accurate estimation on the shear behavior and strength of SFRC members is possible, and the failure mode of steel fibers can be also identified. Four shear behavior models for SFRC members have been proposed, which have been modified from the softened truss models for reinforced concrete members, and they can estimate the contribution of steel fibers to the total shear strength of the SFRC member. The performances of all the models proposed in this study were also evaluated by a large number of test results. The contribution of steel fibers to the shear strength varied from 5% to 50% according to their amount, and the most optimized volume fraction of steel fibers was estimated as 1%–1.5%, in terms of shear performance.

## 1. Introduction

Fiber-reinforced concretes (FRCs) are made with various types of fiber materials, such as steel, carbon, nylon, and polypropylene, which are generally known to have enhanced tensile performance and crack control capability compared to conventional concrete [1,2,3,4,5,6,7]. In particular, it has been reported that steel fibers have an excellent effect on the enhancement of the shear behavior [1,2,3,4,5], and thus, many studies have been conducted on the shear performance of steel-fiber-reinforced concrete (SFRC) members. Most of the previous studies, however, proposed shear strength equations that were empirical based on their experimental results [8,9,10,11,12,13,14], which cannot estimate shear behavior along the loading history of the members, *i.e.*, they cannot provide the shear strains or stresses of the members at a loading stage, except for the ultimate strength. In addition, there are only few shear behavior models for SFRC members, and they mostly modified the tensile stress-strain relationship of concrete to fit for SFRC members. Although they are able to estimate the shear behavior of SFRC members, they cannot identify the strains and stresses in steel fibers, which make it difficult to assess the enhancement of shear performance in detail according to the properties of steel fibers. In this study, therefore, steel fibers were modeled as independent reinforcing materials in the analytical models, and the shape, length, and volume fraction of the steel fibers were reflected in evaluating the shear behavior and strength of SFRC beams. The shear strength models proposed in this study are the smeared crack models that were modified from the softened truss models (STM), which can predict the shear behavior of SFRC members relatively fast, compared to the discrete crack model, by defining the steel fibers on the average that are randomly distributed in concrete without any constant direction. The accuracy of the proposed models was also examined by 85 specimens that were carefully collected from previous studies and by comparison to the shear strength equations proposed by other researchers [9,10,11,12]. In addition, since the proposed models can estimate the stresses in steel fibers, an attempt was also made to evaluate the effectiveness of the steel fibers as a shear reinforcing material by assessing the contribution of the steel fibers to the total shear resistance of SFRC beams.

## 2. Review of Previous Research

### 2.1. Shear Strength Models

In the 1960s, Romualdi and Mandel [15] reported on the tensile strength enhancement of concrete by steel fibers, and Batson *et al.* [16] presented the shear strength enhancement of SFRC beams based on the experimental tests on 102 SFRC beams with the key variables of shear span ratio and volume fraction of steel fibers. Later Swamy and Bahia [17] reported that the shear strength was enhanced due to the steel fibers that deliver the tensile forces at the crack surface in the SFRC beams without shear reinforcement. Sharma [9] performed the experimental study on SFRC beams with the hooked-types of steel fibers, and based on the experiment results, proposed the shear strength (*ν_u_*) equation for the SFRC beams in a relatively simple form, as follows:

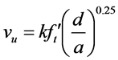
(1)
where *k* is 1 if the tensile strength (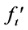) is obtained from a direct tensile test, 2/3 if from a splitting tensile test, and 4/9 if from a flexural tensile test. If Equation (1) is used without tensile tests, 2/3 and 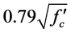 are used for *k* and 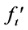, respectively. In addition, *d* is the effective member depth; and *a* is the shear span length. Equation (1) has been used since ACI Committee 544 adopted it in 1988 [1].

Narayanan and Darwish [10] conducted the experiments on SFRC beams, with the primary variables of the splitting tensile strength (*f_sp_*); shear span ratio (*a*/*d*); tensile reinforcement ratio (ρ); fiber coefficient (*F*_1_) and bond strength of steel fibers (τ); and proposed the shear strength (ν*_u_*) equations for SFRC beams, as follows:

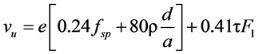
(2)
where *e* is a non-dimensional coefficient considering the arch action, which is 1 for the shear span ratio of greater than 2.8, and 2.8 *d*/*a* for the shear span ratio of less than 2.8. In addition, *F*_1_ is a fiber coefficient that equals to, (*l_f_*/*d_f_*)*V_f_* α where *l_f_*, *d_f_*, and *V_f_* are the length, diameter, and volume fraction of steel fibers, respectively; and α is a bonding coefficient, which is 1.0 for hooked-type fibers, 0.75 for corrugated fibers, and 0.5 for straight fibers.

Ashour *et al.* [8] performed the tests on high-strength SFRC beams, having the compressive strengths of greater than 90 MPa, and proposed the following shear strength (ν*_u_*) equation for the SFRC beams with high-strength concrete:

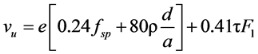
(3)
which is a modified form of the shear strength equation for reinforced concrete (RC) beams presented in the ACI318 [18]. In addition, Ashour *et al.* [8] also proposed the shear strength (*ν_u_*) equations for SFRC members by modifying the Zsutty’s equation[19] for RC beams, as follows:

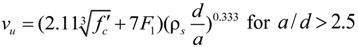
(4)
and

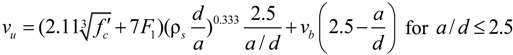
(5)
which consider the shear span ratio (*a*/*d*); tensile reinforcement ratio (ρ*_s_*); fiber coefficient (*F*_1_); and compressive strength (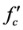). In Equation (5), ν*_b_* is an additional shear resistance by steel fibers in the deep SFRC members, which was recommended as 1.7(*l_f_*/*d_f_*)·*V_f_*·ρ*_f_* based on the Swamy *et al.*’s research [20].

Kwak *et al.* [11] also conducted the experimental study on the SFRC beams, having the compressive strengths of greater than 60 MPa and mixed with hooked-type steel fibers, and proposed the shear strength (*ν_u_*) equation of the SFRC members by adding the term for the contribution of steel fibers into the Zutty’s [19] shear strength equation, as follows:

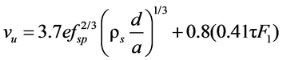
(6)


Oh *et al.* [12] tested the SFRC beams reinforced by angles in tension, instead of reinforcing bars, and proposed the shear strength (*ν_u_*) equation, as follows:

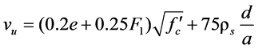
(7)
where *e* is a non-dimensional coefficient considering the arch action, which is 1 for the shear span ratio of greater than 2.5, and 2.5*d*/*a* for the shear span ratio of less than 2.5.

The shear strength equations for SFRC members mentioned [9,10,11,12] here slightly differ from one another, but they are all derived empirically based on test results and mostly include the tensile strength (or compressive strength) of concrete, fiber volume fraction, tensile reinforcement ratio, and shear span ratio as the key influencing parameters. In addition, they have very simplified forms, which are good for their easy application, but, on the other hand, their prediction accuracy can be limited. (Refer to Table 2 and Figure 4 in Chapter 4). Dinh *et al.* [13] proposed a theoretical model for shear strength estimation of SFRC members, in which the shear resistance is calculated by the summation of contributions of the concrete in compression zone and the steel fibers in tension zone. Note that their strength model has not been examined in this paper because its theoretical background is quite different from STM models that authors would like to focus on.

### 2.2. Shear Behavior Models

**Figure 1:**
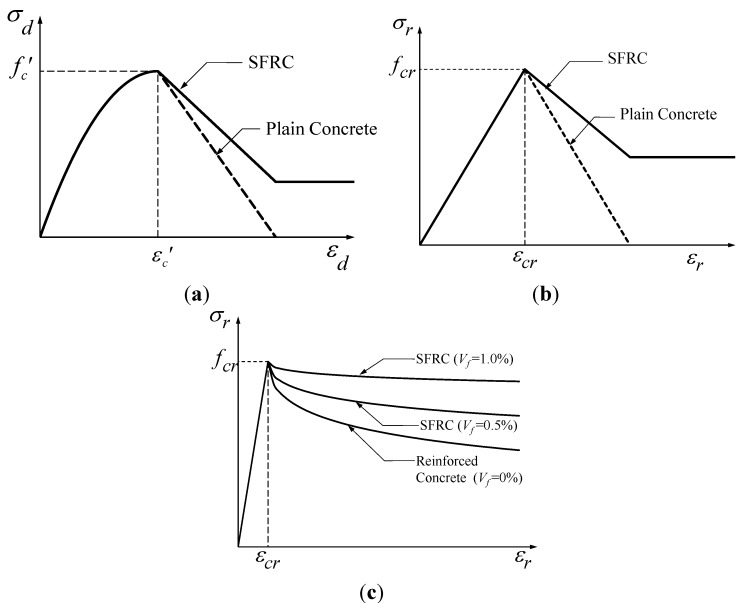
Constitutive models modified by Tan *et al.* (**a**) Compressive stress-strain relationship for rotating angle softened truss models (RA-STM) modified by Tan *et al.* [21]; (**b**) Tensile stress-strain relationship for RA-STM modified by Tan *et al.* [21]; (**c**) Tensile stress-strain relationship for modified compression field theory MCFT modified by Tan *et al.* [23].

Compared to the many equations on the shear strength of SFRC members based on experimental test results, there are only a few studies on the shear behavior models of SFRC members based on analytical research. As shown in Figure 1a,b, Tan *et al.* [21] modified the compression and tension curve of concrete for the rotating angle softened truss model (RA-STM) [22], which took account of the compressive ductility increase and the tension stiffening effect by steel fibers. In other words, his analysis model reflects the effects of steel fibers on the shear behavior of the members through the material curves of SFRC, which is a common modeling for composite materials, and, in fact, provided a good accuracy. It has, however, disadvantages in that it cannot estimate the stresses or strains in the steel fibers, it cannot simulate their residual bond stress or pullout failure, and it cannot count the effects of the fiber volume fraction. Later, Tan *et al.* [23] proposed a shear behavior prediction model that modified the concrete tensile stress-strain relationship for the modified compression field theory (MCFT) [24], as shown in Figure 1c, in which the volume fraction of steel fibers was considered in the tension stiffening effect. As this model was established with insufficient experimental data, it is uncertain whether the volume fraction of steel fibers was properly considered, and other characteristics of steel fibers, such as the shape and length, were not taken into account.

As mentioned, the shear behavior models for SFRC members proposed so far use the stress-strain material curves of SFRC to account for the effect of steel fibers. Thus, they have difficulties in considering the characteristics of steel fibers in details, and cannot consider the failure modes of steel fibers [10,11,25], which often leads to an overestimation of the member ductility. Thus, this study proposed the shear behavior models based on the softened truss models (STM) [22,26,27,28,29,30,31,32], which can estimate the contribution of steel fibers on the shear resistance by modeling them as independent tensile elements, and can simulate their pullout failure modes by reflecting the bond strengths of steel fibers.

## 3. Modified Shear Behavior Models Based on the Softened Truss Models

The shear behavior models of SFRC members proposed in this study are based on four softened truss models, which are summarized here.

### 3.1. Rotating Angle Softened Truss Model (RA-STM)

**Figure 2:**
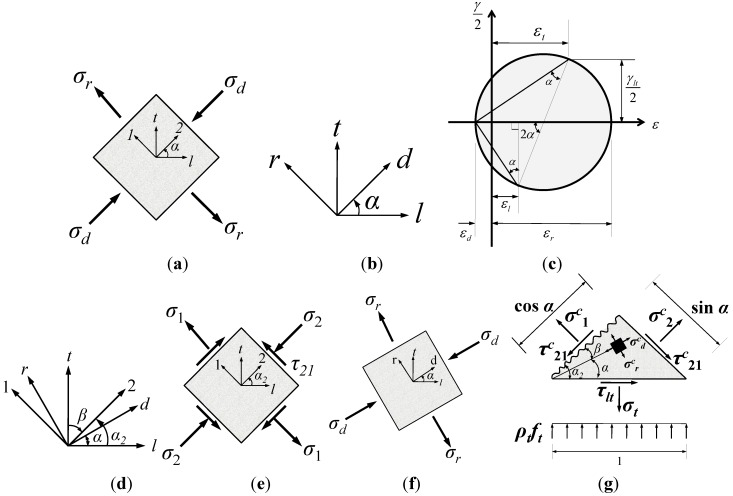
Notations for various softened truss models. (**a**) Stresses in RA-STM; (**b**) Angles in rotated angle model; (**c**) Mohr’s strain circle; (**d**) Angles in fixed angle model; (**e**) Stresses at crack direction; (**f**) Stresses at principal direction; **(g)** Stresses and direction of angles in the transformation angle truss model (TATM).

RA-STM [22,26] is a shear behavior model in which the concrete compression softening and the tension stiffening effect are considered. Since this model is a rotated angle model, wherein the crack angles vary depending on the stress state under the assumption that crack angles are consistent with principal stress angles, the shear stress-strain relationship at the crack is not required. Thus, it is the most simple analysis method for estimating the shear strength and behavior among the four models presented here. Table A1 in Appendix shows the equilibrium, compatibility, and constitutive equations used in RA-STM. As shown in Equation A-1, the horizontal stress, longitudinal stress, and the shear stress can be derived by rotating the stresses in the principal stress direction (*d* − *r* direction) to the direction of *l* − *t* by the principal stress angle (α), as shown in Figure 2a,b. In addition, the compatibility Equation A-2 can be derived using Mohr’s strain circle, as shown in Figure 2c. As for the constitutive equations [33,34], Equation A-3, which considers the compression softening effect, was used for the compressive stress-strain relationship of concrete, and Equation A-4, which reflects the tension stiffening effect, was used for the tensile stress-strain relationship. Equation A-5 was used as the constitutive equations of the longitudinal and shear reinforcements, which considers the hardening phenomenon after the yielding and also the earlier yielding point in a steel bar embedded in concrete compared to the bare bars.

### 3.2. Fixed Angle Softened Truss Model (FA-STM)

As it was assumed, in RA-STM, that the crack direction coincides with the principal stress direction, it was impossible to theoretically consider the shear resistance mechanism at the crack surface, *i.e.*, the aggregate interlock. FA-STM was proposed to solve out such a contradiction in RA-STM. As shown in Figure 2d,e, the shear stresses at the crack surface were considered by fixing the initial crack angle caused by external forces, and the equilibrium equations in FA-STM were derived as shown in Equation A-6 in Appendix. The compatibility equations are also shown in Equation A-7. The constitutive equations of the steel reinforcement and the tensile stress-strain relationship of the concrete are identical to those in RA-STM, but the compressive stress-strain relationship of the concrete was modified to include the reinforcement capacity ratio (η) in the softened coefficient (ζ) as shown in Equation A-3(a and d,f).

The analysis has the following stages. First, before the crack occurs, assume that the crack angle α_2_ by external force is fixed in *2-1* direction. Then, the principal stress angle α of the *d* − *r* direction is determined from the principal stress and the shear stress after cracking, the strains are calculated using the compatibility equations, and the calculated strains are substituted into the constitutive equations to determine the corresponding stresses and the forces. The shear strength can be calculated by iterating the calculation process until the determined forces satisfy the equilibrium condition. In this study, the Zhu *et al.*’s [35] model was used, which is a modified version of the Pang and Hsu’s model [28] that requires more iteration process.

### 3.3. Smeared Membrane Model (SMM)

The Poisson effect could not be considered in the STM mentioned above they were based on the uniaxial strains of concrete. Thus, Hsu and Zhu [36,37] derived the Hsu/Zhu ratio through a panel experiment, which is basically a Poisson ratio, and they implemented it in SMM [30]. SMM is capable of providing the more realistic strains by considering the Poisson effect in the strain compatibility condition. Equation A-14 in the strain compatibility condition gives the equivalent strains in the uniaxial direction considering the Poisson effect by the Hsu/Zhu ratio. The constitutive equations are the same as those in FA-STM, but the shear stress-strain relationship at the crack surface was simplified using the rational shear modulus proposed by Zhu *et al.* [35].

### 3.4. Transformation Angle Truss Model (TATM)

Although the shear stresses at the crack surface seemed to be considered in FA-STM conceptually by fixing the crack angle, most of the analyses by FA-STM actually assumed that the stresses at the crack surface are the same as the principal stresses. Therefore, its application is limited because the difference between the normal stresses (1–2) on the crack surface as shown in Figure 2e and the principal stresss (*d* − *r*) as shown in Figure 2f increases as the difference between the crack angle and the principal stress angle (β) becomes greater. In addition, the constitutive equations in FA-STM were derived from the panel test results, in which the range of the reinforcement capacity ratio was 0.2 < η < 0.5. Thus, it cannot be applied in the cases wherein the reinforcement capacity ratio is below 0.2, which can be often the case in practice. Also, the flexural moment cannot be considered in FA-STM. Thus, Kim and Lee [27,31,32] proposed TATM, modifying FA-STM, in which, as shown in Figure 2g, the principal stresses and strains are obtained by rotating the stresses and strains at the crack surface by β, and the equilibrium equations and the compatibility conditions in the *l* − *t* coordinate system are derived by rotating them again by α. This process requires the shear stress-strain correlation at the crack, for which the equation proposed by Li *et al.* [38] was used, as shown in the first term of A-13(a). In the cases where the axial forces are applied, the Yoshikawa *et al.*’s equation [39], as shown in the second term of Equation A-13(a), was superimposed. In addition, in order to consider the flexural moment effect, the steel ratio required to resist the flexure was subtracted, and the remained reinforcement ratio was assumed to resist the shear.

## 4. Proposed Model: Softened Truss Model with Steel Fibers (STM-SF)

**Figure 3:**
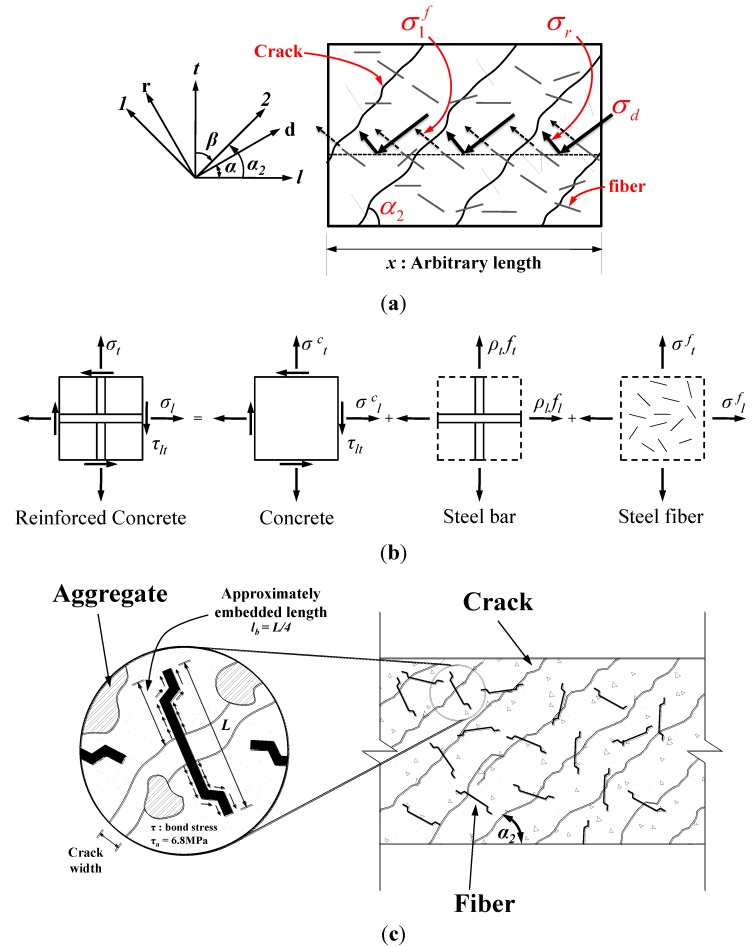
Description of the proposed model for SFRC members. (**a**) Description of steel fibers in cracked concrete; (**b**) Equilibrium in a SFRC element; (**c**) Bonded length of a steel fiber at crack.

In this study, steel fibers are considered as independent reinforcement materials, and it is assumed that a certain number of steel fibers, which are distributed randomly according to the fiber volume fraction, resist the tensile stress perpendicular to the crack surface, as shown in Figure 3a [4]. In addition, steel fibers are assumed to show full composite behavior with concrete before the pull-out of steel fibers occurs, from which, the strains of steel fibers can be considered to be the same as the average strains of concrete at the same location. As shown in Figure 3b, the tensile resistances of steel fibers are added to the equilibrium conditions of the softened truss models in the normal direction. Thus, the additional term by the steel fibers in the equilibrium equations in the *l* − *t* direction can be derived by rotating the stress of the steel fibers at the crack surface by the crack angle (α_2_), as follows:

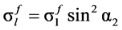
(8)

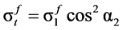
(9)

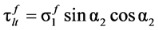
(10)
where α_2_ is the crack angle; and 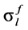, 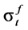, and 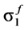 are the average stresses of steel fibers in the longitudinal direction, in the transverse direction, and in the crack direction, respectively. Thus, the final forms of the equilibrium equations for SFRC members can be obtained by adding Equations (8)–(10) to the equilibrium equations of RA-STM, FA-STM, SMM, and TATM in the longitudinal and transverse directions.

The stress-strain relationship of steel fibers can be expressed, assuming their elastic-plastic behavior, as follows:

σ*_f_* = *E_f_*ε_1_ ≤ *f_yf_* (11)

where σ*_f_* is the stress of steel fibers; *f_yf_* is the yield strength; *E_f_* is the elastic modulus and 200 GPa can be used [40], and ε_1_ is the tensile stress at the crack surface.

The tensile force resisted by the steel fibers (*T_f_*) can be calculated by multiplying the number of the steel fibers on the crack plane (*n*) by their tensile stress (σ*_f_*) and their cross-sectional areas (*A_f_*), as follows:

*T_f_* = σ*_f_nA_f_* (12)

Then, the average tensile stress (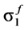) of the steel fibers on the crack plane can be expressed by dividing the tensile force (*T_f_*) by the area of the crack surface (*A_cs_*), as follows:

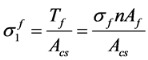
(13)


In Equation (13), the number of the steel fibers on the crack plane (*n*) can be determined by multiplying the number of the steel fibers on the crack surface per unit area (*n_w_*) by the area of the crack surface (*A_cs_*), as follows:

*n* = *n_w_A_cs_* (14)

Romualdi *et al.* [15] proposed the number of the steel fibers on the crack surface per unit area (*n_w_*) considering the orientation of the steel fibers, which was adopted in this study, as follows:

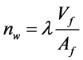
(15)
where *V_f_* is the volume fraction of the steel fibers, and λ is the directional coefficient that considers the orientation of the steel fibers, for which 0.41 is used in this study as recommended by Romualdi *et al.* [15]. Then, by substituting the number of steel fibers on the crack surface (*n*) in Equations (14) and (15) to that in Equation (13), the average tensile stress (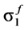) of the steel fibers on the crack surface can be rearranged as follows:

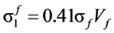
(16)

When the fiber stress (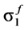) reaches its maximum bond stress, the pullout failure of the steel fibers would occur. Thus, the maximum value of the fiber stress (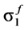) should be limited to the maximum bond stress (τ*_max_*), and accordingly, the pullout strength (σ*_fp_*) of steel fibers can be derived as follows:

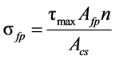
(17)
where *A_fp_* is the average surface area of steel fibers, on which the bond stress is developed, and the maximum bond stress (τ_max_) can be calculated as follows:

τ_max_ = τ*_u_d_f_* (18)

where τ*_u_* is the bond strength of hooked-type fibers, for which 6.8 MPa is used in this study as proposed by Lim *et al.* [40]; and *d_f_* is the shape factor of steel fibers, for which Narayanan and Darwish [10] proposed 1.0 for hooked-type fibers, 0.75 for crimp-type fibers, and 0.5 for straight type fibers. Therefore, the ultimate bond strength of steel fibers (σ*_fp_*) in an average sense, considering their shapes and the corresponding maximum bond stress (τ_max_), can be summarized as follows:

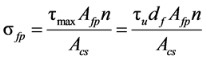
(19)


The steel fibers are randomly distributed and typically short compared to the member size, the embedded lengths (*l_b_*) of the steel fibers at cracking cannot be determined accurately. Accordingly, as shown in Figure 3c, it is assumed that one-fourth of the fiber length is the average bond length. Then, Equation (19) can be modified by as follows [10]:

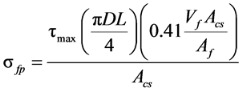
(20)
where *D* and *L* are the diameter and length of a steel fiber, respectively. The pullout strength of steel fibers or the average ultimate bond strength (σ*_fp_*) can be further simplified from Equation (20), as follows:

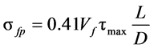
(21)


Accordingly, the equilibrium equations for SFRC members, including the tensile resistance of steel fibers, can be expressed as follows:

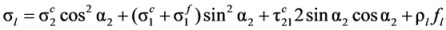
(22)

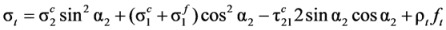
(23)

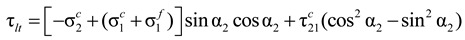
(24)


The compatibility equations and constitutive relationships of materials are used as in each softened truss model, shown in Appendix. In addition, the SFRC member is considered to reach its maximum strength either when the pull out failure of steel fibers occurs or when the principal compressive strain (ε*_d_*) reaches the maximum strain of concrete (ζε_0_), the SFRC member is considered reach their maximum strength.

## 5. Evaluation of the Proposed Models

For the purpose of evaluation on the shear behavior models proposed in this study, the shear test results of SFRC beams has been collected from literature [2,8,10,16,25,41,42,43,44], as shown in Table 1. Of the total of 132 specimens collected, the specimens that had flexural failures or that were deep beams with a shear span-to-depth ratio (*a*/*d*) of 2.5 or less were excluded, and thus, a total of 85 shear specimens was used in this study. The steel fiber volume fraction of the collected specimens ranged from 0.22% to 2.0%, and the size of steel fibers used in the specimens ranged widely from the small ones with the length of 25.4 mm and the diameter of 0.25 mm to the big ones with the length of 60 mm and the diameter of 0.8 mm. In addition, the steel fibers included straight, crimped and hooked types. The concrete compressive strengths (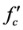) also ranged widely from 20.6 to 93.8 MPa, including normal-strength concrete and high-strength concrete. All the specimens that were used for the evaluation did not have shear reinforcements, and the tensile steel ratio (ρ*_s_*) ranged from 1.1% to 5.7%.

**Figure 4:**
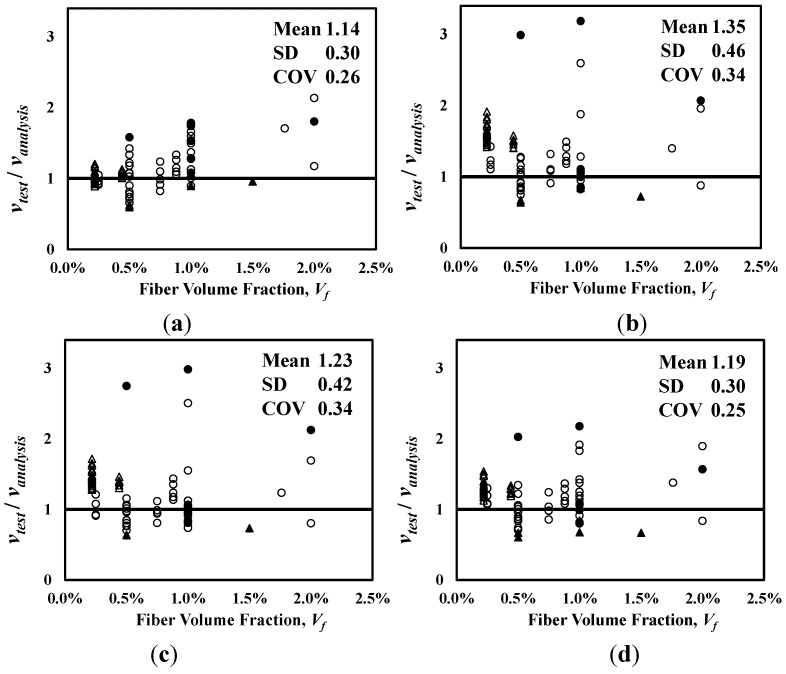
Comparison of analysis results. (**a**) Sharma [9]; (**b**) Narayanan and Darwish [10]; (**c**) Kwak *et al.* [11]; (**d**) Oh *et al.* [12]. ●: 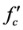 ≥ 50 MPa, *a*/*d* < 4; ○: 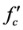 < 50 MPa, *a*/*d* < 4; ▲: 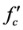 ≥ 50 MPa, *a*/*d* ≥ 4; △: 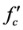 < 50 MPa, *a*/*d* ≥ 4.

Figure 4 shows the analysis results of the shear strength equations presented in Equations (1), (2), (5), and (6), which are also summarized in Table 2 with other analysis results. In Figure 4a–d, the vertical axis represents the ratio of the test results to the analysis results (*ν_te__s__t_*/*ν_anal__y__sis_*), and the horizontal axis represents the fiber volume fraction. Also, the mean, standard deviation (SD) and coefficient of variation (COV) of the *ν_te__s__t_*/*ν_anal__y__sis_* values are presented in each graph. The equation proposed by Sharma [9], which has been adopted by the ACI Committee 544 [1], and the one recently proposed by Oh *et al.* [12] showed relatively good accuracy with the low COVs of 0.26 and 0.25, respectively. The equations proposed by Narayanan and Darwish [10] and Kwak *et al.* [11] are, however, showed a large scatter, especially for the specimens cast with normal-strength concrete.

**Table 1:** Dimensions and properties of SFRC specimens.

Reference No.	Number of specimens	shape	*V_f_* (%)	*L* (mm)	*D* (mm)	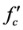 (MPa)	*d* (mm)	*a*/*d*	ρ*_s_* (%)
[16]	13	round	0.22–0.44	25.4	0.25	33.2–40.2	127	4.0–4.8	1.96
	24	crimped	0.22–1.76	25.4	0.25 × 5.6 * 0.38 × 0.63* 0.41 × 0.25*	33.2–40.2	127	4.0–4.8	1.96
[10]	18	crimped	0.25–1.0	30–40	0.3	29.9–59.6	126–130	2.5–3.5	2.00–5.72
[2]	7	hooked	0.5–1.0	30	0.5	20.6–33.4	197	2.8–3.6	1.34–2.00
[25]	5	hooked	0.5–1.0	30	0.5	34	221	2.5–3.5	1.10–2.20
[8]	5	hooked	0.5–1.5	60	0.8	93.8–97.1	215	4.0–6.0	2.84–4.58
[41]	5	hooked	1.0	30–50	0.5	22.7–26	102–204	3.0	1.10–2.20
[42]	4	crimped	0.5–2.0	25.4–38.1	0.2 × 2.3*	49.3–54.8	80	3.75	1.77
[43]	2	round	1.0–2.0	42	0.7	38.7–42.4	150	2.67	2.65
[44]	2	hooked	1.0–2.0	30	0.5	40.9–43.2	219	2.8	1.74
Total	85	round, crimped, hooked	0.22–2.0	25.4–60	0.25–0.8	20.6–97.1	80–221	2.5–6.0	1.10–5.72

* Rectangular Section

**Figure 5:**
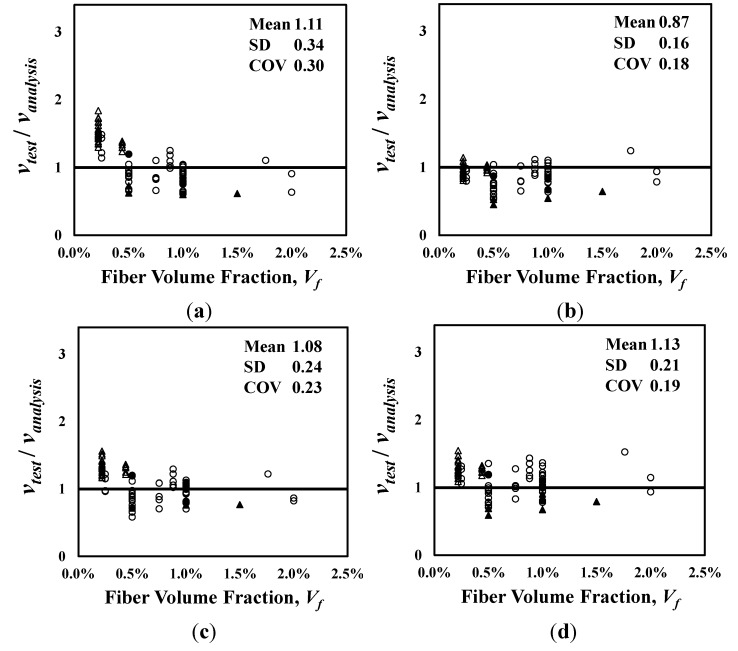
Verification of the proposed models. (**a**) The modified RA-STM with steel fibers; (**b**) The modified FA-STM with steel fibers; (**c**) The modified TATM with steel fibers; (**d**) The modified SMM with steel fibers. ●: 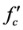 ≥ 50 MPa, *a*/*d* < 4; ○: 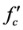 < 50 MPa, *a*/*d* < 4; ▲: 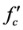 ≥ 50 MPa, *a*/*d* ≥ 4; △: 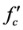 < 50 MPa, *a*/*d* ≥ 4.

Figure 5 shows the analysis results of the softened truss models with steel fibers (STM-SF) proposed in this study, which are also summarized in Table 2 with other analysis results. Note that, while Figure 5 shows the *ν_te__s__t_*/*ν_anal__y__sis_* values *versus* the fiber volume fraction in the graph, it also gives the data ranges in terms of the compressive strength and the shear span-depth ratio, as indicated at the bottom of the graphs. As shown in Figure 5a, the modified RA-STM with steel fibers provided a mean of 1.11 and a COV of 0.30, which was a relatively larger scatter compared to the other STM-SF analysis models. This model tended to overestimate the specimens with high-strength concrete, and was relatively inaccurate for the specimens with low steel fiber volume fractions. The principal stress angle is assumed to be identical with the crack angle in RA-STM, but their difference becomes bigger in the specimens with a low steel fiber volume fraction [45], which leads to underestimate the tensile resistance of the steel fibers on the crack surface in such cases. The modified FA-STM with steel fibers showed a relatively high accuracy, with a mean of 0.87 and a COV of 0.18, as shown in Figure 5b, and there was no bias in the *ν_te__s__t_*/*ν_anal__y__sis_* values. However, this model tended to overestimate, in particular, the specimens with a high shear span-depth ratio, which seems to be because FA-STM cannot consider the flexural moment effects.

**Table 2:** Comparison of analysis results.

Model	RA-STM with steel fiber	FA-STM with steel fiber	TATM with steel fiber	SMM with steel fiber
Mean	1.112	0.871	1.082	1.131
SD	0.338	0.157	0.244	0.213
COV	0.304	0.181	0.225	0.188
**Author**	Sharma (ACI) [9]	Narayanan *et al.* [10]	Kwak *et al.* [11]	Oh *et al.* [12]
Mean	1.143	1.345	1.229	1.188
SD	0.298	0.456	0.419	0.296
COV	0.261	0.339	0.341	0.249

The modified TATM with steel fibers, as shown in Figure 5c, provided a good accuracy, with a mean of 1.08 and a COV of 0.23. In particular, this model provided more reasonable analysis results for the cases with large shear span ratios (*a*/*d*), which is considered to be because this model can take account of the flexural moment effect. In addition, this model can reflect the difference between the crack angle and the principal stress angle (β), which indeed improved the analysis accuracy in overall. The analysis results of the modified SMM with steel fibers are shown in Figure 5d. It provided a high accuracy with a COV of 0.19, and had no bias along the volume fractions of steel fibers. The improved accuracy in this model seems to come from the consideration of the Poisson effect, and it could be even more accurate if the Poisson ratio after cracking could be obtained from SFRC panel experiments [46].

Overall, all the modified STM models, except the modified RA-STM with steel fibers, provided a good level of accuracy on the shear strength of SFRC members, which implies that the characteristics of steel fibers are well reflected in these models proposed in this study. The existing empirical equations showed relatively larger scatter for those test results that were not within the variable ranges included at the time of their formulation. It is also worth noting that the proposed models are based on the Smeared Crack Model [22,26,27,28,29,30,31,32] that uses the average stress and average strain relationship, and that they successfully simulate the shear failure modes of SFRC beams, *i.e.*, the pullout failure of steel fibers considering their bond strengths.

**Figure 6:**
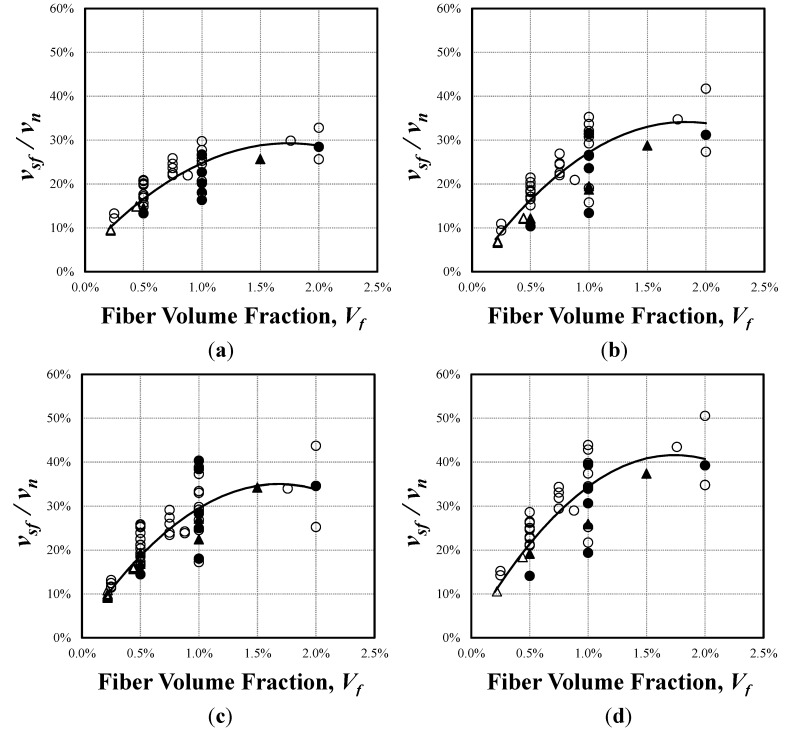
Contribution of steel fibers to shear strength at failure. (**a**) The modified RA-STM with steel fibers; (**b**) The modified FA-STM with steel fibers; (**c**) The modified TATM with steel fibers; (**d**) The modified SMM with steel fibers. ●: 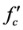 ≥ 50 MPa, *a*/*d* < 4; ○: 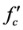 < 50 MPa, *a*/*d* < 4; ▲: 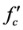 ≥ 50 MPa, *a*/*d* ≥ 4; △: 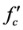 < 50 MPa, *a*/*d* ≥ 4.

As aforementioned, the contribution of steel fibers to the total shear resistance can be estimated by the proposed models because the steel fibers are modeled as an independent tensile element. Figure 6 presents the contribution of steel fibers to the shear resistance (*ν_sf_*/*ν_n_*) at ultimate according to fiber volume fractions (*V_f_*), where *ν_n_* is the calculated shear strength and the shear resistance of steel fibers (*ν_sf_*) is calculated from Mohr’s stress circle, as follows:

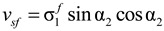
(25)


In all the analysis models, the shear contribution of the steel fibers increased as the steel fiber volume fractions increased. In the modified RA-STM with steel fibers, the shear contribution ratio of steel fibers (*ν_sf_*/*ν_lt_*) was calculated as approximately 10% at the lowest fiber volume fraction of 0.22%, and as high as 30% at the maximum fiber volume fraction of 2%. In addition, the increase rate of the shear contribution ratio of steel fibers significantly changes at 1%–1.5% steel fiber volume fractions, and it becomes almost flat at 1.5%–2.0% steel fiber volume fractions. The modified FA-STM with steel fibers showed shear contribution ratio similar to that of the modified RA-STM for the SFRC members with the low fiber volume fractions, but demonstrated higher shear contribution ratios for those with the volume fractions of 1% or higher. Also, the shear contribution ratio of steel fibers showed a considerable variation at the volume fraction of 1%. The modified TATM with steel fibers provided very close results to the modified FA-STM, which showed the shear contribution ratio of approximately 30% at 1%–1.5% steel fiber volume fractions. The modified SMM with steel fibers showed higher shear contribution ratios of steel fibers than other models, in which the contribution ratios ranged from 10 to 50%. The model showed a significant variation at the 1% volume fraction, similar to FA-STM, and the increase in the shear contribution ratio of steel fibers also dropped at the 1%–1.5% fiber volume fractions.

The observations above confirm the substantial contribution of steel fibers to the improvement of the shear strength of SFRC members, and it is also clear that the steel fiber volume fraction is the key influencing parameter on the shear strength of SFRC members. The shear contribution ratios of steel fibers ranged from 8% to 45% at the steel fiber volume fractions below 1%, and it ranged from 13%–50% at the steel fiber volume fractions over 1%. It was also found that the increase rate of the steel fiber contribution significantly reduced at 1%–1.5% steel fiber volume fractions, and that it was almost flat at 1.5%–2.0% steel fiber volume fractions. This is because the inclined compression strut of concrete first reaches at failure, even if the steel fiber volume fraction increases. Therefore, the optimal volume fraction ratio in terms of shear performance appears to exist between 1% and 1.5%, which is also consistent with the observations in previous studies [47,48].

## 6. Conclusions

Most of shear strength equations for SFRC members are relatively simple, but provide a low accuracy, as they have been derived empirically based on experimental test results. Some analytical models can estimate shear behavior and strength of SFRC members, but cannot provide the contribution of steel fibers to the shear strength and cannot demonstrate the pullout failure of steel fibers. In this study, the softened truss models were modified appropriately for SFRC members, in which the steel fibers were modeled as independent tensile elements so that the proposed models can reflect the details of steel fibers such as the effects of the shape, length, and volume fraction of steel fibers. The proposed models were also compared to the test results of 85 specimens collected from literature. From this study, the following conclusions were drawn.

1. The softened truss models were modified to be suitable for the analysis of SFRC members by modeling steel fibers as independent tensile elements, which, in particular, can estimate the stresses of steel fibers according to the detailed characteristics of the steel fibers.
2. All the STM-SF models proposed in this study, except for the modified RA-STM with steel fibers, showed a good level of accuracy on the shear strength of SFRC members compared to the empirical equations presented in previous studies.
3. The proposed models adequately simulated the pullout failure of steel fibers, which is the characteristic failure mode in SFRC members, based on the average ultimate bond strength of steel fibers.
4. The modeling method, applying the stress of fibers perpendicular to crack direction directly, was considered more appropriate in FASTM than RASTM; it is, because, as expected, the fixed angle model could reflect the stress of fibers at crack more accurately.
5. The contribution ratios of steel fibers on the shear strength of SFRC members were calculated by the proposed models, which was found to be approximately 30% at the 1%–1.5% steel fiber volume fraction.
6. Based on the observations of the shear contribution ratio of steel fibers, the optimal range of the steel fiber volume fraction, in terms of shear performance, is 1%–1.5%.

## Appendix

**Table A-1:** Equations in RA-STM and FA-STM.

Model	RA-STM		FA-STM	
Equilibrium equations		(A-1a)		(A-6a)
		(A-1b)		(A-6b)
		(A-1c)		(A-6c)
Comparability equations		(A-2a)		(A-7a)
		(A-2b)		(A-7b)
		(A-2c)		(A-7c)
Constitutive equations	**Concrete compression**		**Concrete compression**	
		(A-3a)		(A-3a)
		(A-3b)		(A-3d)
		(A-3c)		(A-3e)
	**Concrete tension**			(A-3f)
		(A-4a)	**Concrete tension**	
		(A-4b)		(A-4a)
	**Mild steel**			(A-4b)
		(A-5a)	**Mild steel**	
		(A-5b)		(A-5a)
		(A-5c)		(A-5b)
		(A-5d)		(A-5c)
				(A-5d)
			Shear stress of concrete at crack	
				(A-8)

**Table A-2:** Equations in TATM and SMM.

Model	SMM		TATM	
Equilibrium equations				(A-6a)
				(A-6b)
		(A-6a)		(A-6c)
		(A-6b)		(A-9a)
		(A-6c)		(A-9b)
				(A-9c)
Comparability equations		(A-14a)		
		(A-14b)		(A-7a)
		(A-14c)		(A-7b)
		(A-14d)		(A-7c)
		(A-7c)		
Constitutive equations	**Concrete compression**		**Concrete compression**	
		(A-3a)		(A-10a)
		(A-3d)		(A-10b)
		(A-3e)	**Concrete tension**	
				(A-11a)
		(A-3f)		(A-11b)
	**Concrete tension**		**Steel**	
		(A-4a)		(A-12a)
		(A-4b)		(A-12b)
	**Mild steel**		**Shear stress of concrete at crack**	
		(A-5a)		(A-13a)
		(A-5b)		(A-13b)
		(A-5c)		(A-13c)
		(A-5d)		(A-13d)
	**Shear stress of concrete at crack**			(A-13e)
		(A-8)		
